# Cardiovascular outcomes trials: a paradigm shift in the current management of type 2 diabetes

**DOI:** 10.1186/s12933-022-01575-9

**Published:** 2022-08-04

**Authors:** Melanie J. Davies, Heinz Drexel, François R. Jornayvaz, Zoltan Pataky, Petar M. Seferović, Christoph Wanner

**Affiliations:** 1 Diabetes Research Centre, University of Leicester, Leicester, UK; 2 NIHR Leicester Biomedical Research Centre, Leicester, UK; 3 University Hospitals of Leicester NHS Trust, Leicester, UK; 4 Vorarlberg Institute for Vascular Investigation and Treatment (VIVIT), Landeskrankenhaus Feldkirch, Feldkirch, Austria; 5 Service of Endocrinology, Diabetes, Nutrition and Therapeutic Patient Education, WHO Collaborating Centre, Geneva University Hospital/Geneva University, Geneva, Switzerland; 6 University of Belgrade, Faculty of Medicine, Belgrade, Serbia; 7 Serbian Academy of Sciences and Arts, Belgrade, Serbia; 8 Würzburg University Clinic, Würzburg, Germany

**Keywords:** Cardiovascular disease, Cardiovascular outcomes trials, Chronic kidney disease, CVOTs, Cardiovascular safety, Heart failure, Glucose-lowering drug, GLP-1 RAs, Type 2 diabetes, SGLT2 inhibitors

## Abstract

Cardiovascular disease (CVD) is the leading cause of mortality and morbidity in patients with type 2 diabetes (T2D). Historical concerns about cardiovascular (CV) risks associated with certain glucose-lowering medications gave rise to the introduction of cardiovascular outcomes trials (CVOTs). Initially implemented to help monitor the CV safety of glucose-lowering drugs in patients with T2D, who either had established CVD or were at high risk of CVD, data that emerged from some of these trials started to show benefits. Alongside the anticipated CV safety of many of these agents, evidence for certain sodium–glucose transporter 2 (SGLT2) inhibitors and glucagon-like peptide-1 receptor agonists (GLP-1 RAs) have revealed potential cardioprotective effects in patients with T2D who are at high risk of CVD events. Reductions in 3-point major adverse CV events (3P-MACE) and CV death have been noted in some of these CVOTs, with additional benefits including reduced risks of hospitalisation for heart failure, progression of renal disease, and all-cause mortality. These new data are leading to a paradigm shift in the current management of T2D, with international guidelines now prioritising SGLT2 inhibitors and/or GLP-1 RAs in certain patient populations. However, clinicians are faced with a large volume of CVOT data when seeking to use this evidence base to bring opportunities to improve CV, heart failure and renal outcomes, and even reduce mortality, in their patients with T2D. The aim of this review is to provide an in-depth summary of CVOT data—crystallising the key findings, from safety to efficacy—and to offer a practical perspective for physicians. Finally, we discuss the next steps for the post-CVOT era, with ongoing studies that may further transform clinical practice and improve outcomes for people with T2D, heart failure or renal disease.

**Supplementary Information:**

The online version contains supplementary material available at 10.1186/s12933-022-01575-9.

## Introduction

The prevalence of type 2 diabetes (T2D) has continued to rise over recent years. It is estimated that by 2045 there will be 693 million people diagnosed with the condition worldwide [1]. T2D poses significant health risks to individuals, with a two-fold increase in mortality compared with a population without diabetes [2], as well as an increasing global health economic burden [3]. Associations between T2D and cardiovascular disease (CVD) are well established; CVD is the leading cause of mortality and morbidity in patients with T2D [2–4], and more than 30% of patients with T2D are diagnosed with CVD [4]. The most common CVD manifestations in patients with T2D are peripheral arterial disease, ischaemic stroke, stable angina, heart failure (HF) and nonfatal myocardial infarction (MI) [3, 5]. A recent meta-analysis showed that patients with coexisting diabetes and HF have an increased risk of all-cause death, cardiovascular (CV) death and hospitalisation [6]. Moreover, one in six patients with newly diagnosed T2D have evidence of silent MI associated with an increased risk of all-cause mortality (HR 1.26, 95% CI 1.06–1.50) and fatal MI (HR 1.49, 95% CI 1.15–1.94) [7]. Reducing CV risk is a key part of T2D disease management [3].

**Fig. 1:**
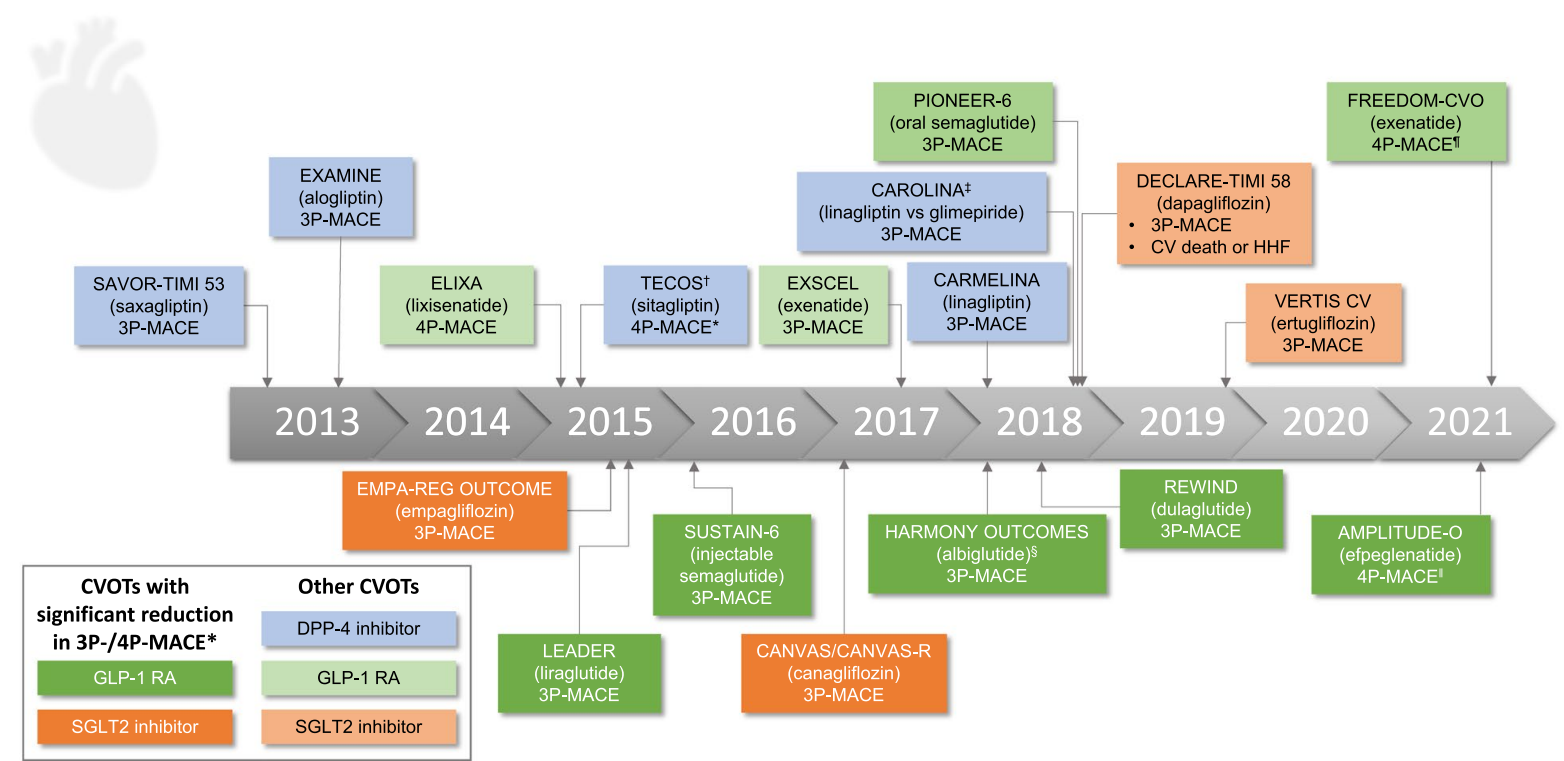
A timeline of published diabetes CVOTs. The comparator in all trials was placebo, unless otherwise stated. Primary endpoints for each trial are listed. 3/4P-MACE, 3/4-point major adverse CV event; CV, cardiovascular; DPP-4, dipeptidyl peptidase-4; GLP-1 RA, glucagon-like peptide-1 receptor agonist; HHF, hospitalisation for heart failure; SGLT2, sodium–glucose transporter 2. Source: clinicaltrials.gov. *3P-MACE is a composite of CV death, nonfatal myocardial infarction and nonfatal stroke. 4P-MACE is an expanded composite of 3P-MACE plus either hospitalisation for unstable angina (ELIXA, TECOS and FREEDOM-CVO) or death from undetermined causes (AMPLITUDE-O). ^†^TECOS and FREEDOM-CVO included 3P-MACE as a secondary outcome. ^‡^CAROLINA was conducted in addition to regulatory requirements, as an active-controlled CVOT complementary to the core placebo-controlled CVOT CARMELINA. ^§^Albiglutide is no longer a licensed treatment. ^‖^Efpeglenatide is not a currently licensed treatment. ^¶^FREEDOM-CVO (exenatide subcutaneous implant; not a currently licensed treatment) was completed in 2016, but the primary outcome (4P-MACE) was reported in 2022

Until around a decade ago, the standard of care for T2D involved the use of glucose-lowering drugs (GLDs) such as metformin, sulfonylureas, thiazolidinediones, meglitinides and α-glucosidase inhibitors [8]. However, amid uncertainty about the CV safety of GLDs [9–12], in 2008 the U.S. Food and Drug Administration (FDA) updated its guidance, mandating the assessment of all new T2D therapies in long-term CV outcomes trials (CVOTs), in addition to the requirement for registrational studies demonstrating improvements in glycaemic control [13]. In the meantime, newer GLD classes have become firmly established treatments for T2D, i.e. dipeptidyl peptidase-4 (DPP-4) inhibitors, glucagon like peptide-1 receptor agonists (GLP-1 RA) and sodium–glucose cotransporter-2 (SGLT2) inhibitors. To date, 18 CVOTs have been published for these newer GLDs (Fig. 1), which enrolled patients with T2D who had established CVD or were at high risk of CVD [13–24], and had to demonstrate a hazard ratio (HR) < 1.8 for major CV events (MACE; based on the upper bound of a two-sided 95% confidence interval [CI]). Most CVOTs included the key composite outcome of 3-point MACE (3P-MACE; comprising CV death, nonfatal MI and nonfatal stroke), with the exceptions of additional events in a 4P-MACE in the ELIXA trial of lixisenatide (hospitalisation for unstable angina) and in the AMPLITUDE-O trial of efpeglenatide (death from undetermined causes) [10, 25, 26]. Notably, some CVOTs have not only illustrated CV safety, but also reported cardioprotective benefits. The first of these was EMPA-REG OUTCOME, completed in 2015, which showed that the SGLT2 inhibitor empagliflozin reduced 3P-MACE and CV death in patients with T2D and established CVD [27]. Hospitalisation for heart failure (HHF), all-cause mortality and progression of kidney disease were also reduced with empagliflozin [27–29]. Subsequently published CVOTs, as well as a small number of HF and renal outcomes studies, have added further paradigm-shifting evidence for improvements in CV, HHF and renal outcomes during treatment with other GLDs, such as the SGLT2 inhibitor canagliflozin, in patients with T2D (Table 1; Additional file 1: Table S1) [15, 16, 27, 30–37]. CVOT findings are now a major focus of updated treatment guidelines (Table 2) [38–44] and product labels [13].

**Table 1:** Overview of CVOTs reporting significant reductions in 3P/4P-MACE

Class*^†^	SGLT2 inhibitors		GLP-1 receptor agonists			
**Study**	EMPA-REG OUTCOME Empagliflozin	CANVAS Programme Canagliflozin	AMPLITUDE-O Liraglutide	LEADER Liraglutide	SUSTAIN-6 Semaglutide	REWIND Dulaglutide
**MACE**^**‡**^ HR (95% CI) **ER** drug vs placebo/1,000 PY	0.86 (0.74–0.99) 37 vs 44	0.86 (0.75–0.97) 27 vs 32	0.73 (0.58–0.92) 39 vs 53	0.87 (0.78–0.97) 34 vs 39	0.74 (0.58–0.95) 32 vs 44	0.88 (0.79–0.99) 24 vs 27
**CV death** HR (95% CI)	0.62 (0.49–0.77) All-cause death also reduced	0.87 (0.72–1.06)–*N.S*	0.72 (0.50–1.03)–*N.S*	0.78 (0.66–0.93) All-cause death also reduced	0.98 (0.65–1.48)–*N.S*	0.91 (0.78–1.06)–*N.S*
**Nonfatal MI** HR (95% CI)	0.87 (0.70–1.09)–*N.S*	0.85 (0.69–1.05)–*N.S*	0.78 (0.55–1.10)–*N.S*	0.88 (0.75–1.03)–*N.S*	0.74 (0.51–1.08)–*N.S*	0.96 (0.79–1.16)–*N.S*
**Nonfatal stroke** HR (95% CI)	1.24 (0.92–1.67)–*N.S*	0.90 (0.71–1.15) – *N.S*	0.80 (0.48–1.31) – *N.S*	0.89 (0.72–1.11) – *N.S*	0.61 (0.38–0.99)	0.76 (0.61–0.95)
**Other cardiorenal benefits** (individual secondary endpoints)	Protective effect on: • HHF • Impaired renal function • Albuminuria	Protective effect on: • HHF • Impaired renal function • Albuminuria	Protective effect on: • HF • A composite of impaired renal function or albuminuria • Albuminuria	Protective effect on albuminuria	Protective effect on albuminuria	Protective effect on: • Impaired renal function • Albuminuria
*Cohort composition*						
**Number of participants**	7020	10,142	4076	9340	3297	9901
**Established CVD** % pts	99%	65%	91%	82%	83%	31%
**Mean eGFR** mL/min/1.73 m^2^	74	77	73	80	76	75
**Key inclusion criteria** (in addition to T2D)	Age ≥ 18 years with established CVD	• Age ≥ 30 years with symptomatic ASCVD • or ≥ 50 years with ≥ 2 CV risk factors	• Age ≥ 18 years with history of CVD • or ≥ 50 years (male) or ≥ 55 years (female) with kidney disease and ≥ 1 CV risk factor	• Age ≥ 50 years with ≥ 1 CV condition • or ≥ 60 years with ≥ 1 CV risk factor	• Age ≥ 50 years with established CVD, chronic HF or chronic kidney disease (> stage 3) • or ≥ 60 years with ≥ 1 CV risk factor	• Age ≥ 50 years with vascular disease • or ≥ 55 years with ≥ 1 cardiorenal condition • or ≥ 60 years with ≥ 2 CV risk factors
*Subgroup analyses*						
**Secondary vs primary CVD prevention** **MACE**^**‡**^ HR (95% CI)	N/A	Secondary prevention group: 0.82 (0.72–0.95) Primary prevention group: 0.98 (0.74–1.30) P = 0.18	Secondary prevention group: 0.71 (0.57–0.90) Primary prevention group: 1.71 (0.48–6.07)	Secondary prevention group: 0.83 (0.74–0.93) Primary prevention group: 1.20 (0.86–1.67) P = 0.04	Secondary prevention group: 0.72 (0.55–0.93) Primary prevention group: 1.00 (0.41–2.46) P = 0.49	Secondary prevention group: 0.87 (0.74–1.02) Primary prevention group: 0.87 (0.74–1.02) P = 0.97
**Other subgroups**	Relative risk reduction for 3P-MACE was in most cases broadly similar across demographic and clinical baseline characteristics, including a range of cardiovascular and renal characteristics					

(For detailed overview of all diabetes CVOTs, and renal outcomes and HF studies, see Additional file 1: Table S1). Primary and key secondary endpoints, patient cohort composition, and key subgroup analyses for glucose-lowering agents that have demonstrated ASCVD benefits in diabetes CVOTs [15, 27, 28, 31, 32, 34, 96, 97]. As most CVOTs were not head-to-head trials, direct comparisons of agents cannot be made, due to possible differences in study design, definitions and cohorts. For example, absence of a demonstrated benefit may be due to such factors, especially for secondary outcomes where studies may not be powered to reach statistical significance. Differences in baseline CV risk are substantial between CVOTs, and even the definition of CV risk and individual risk factors differs between trials. CVOTs excluded here include: DECLARE-TIMI 58 (dapagliflozin), which did not show a significant effect on 3P-MACE, and a reduced risk for the composite of CV death or HHF was driven by reduction in HHF [37]; EXSCEL (once-weekly exenatide) found a non-significant trend towards a reduction of 3P-MACE [33]. References: EMPA-REG OUTCOME [27–29, 62]; CANVAS Program [30, 62, 92]; AMPLITUDE-O [23]; LEADER [32, 96]; SUSTAIN-6 [31, 136–138]; REWIND [34, 97]

3P/4P-MACE, 3-/4-point major adverse cardiovascular event; ASCVD, atherosclerotic CVD; CI, confidence interval; CKD, chronic kidney disease; CV, cardiovascular; CVD, CV disease; CVOT, CV outcomes trial; eGFR, estimated glomerular filtration rate; ER, event rate; GLP-1, glucagon-like peptide 1; HR, hazard ratio; MI, myocardial infarction; N.S., not significant; PY, patient-years; SGLT2, sodium–glucose transport protein 2; T2D, type 2 diabetes

*All drugs shown are currently licensed for T2D, except for efpeglenatide

^†^In addition to the CVOTs shown, the HARMONY OUTCOMES trial showed reduced risks of 3P-MACE and MI with the GLP-1 receptor agonist albiglutide [35]; however, albiglutide is no longer an approved treatment

^‡^3P-MACE is shown for all CVOTs (a composite of CV death, nonfatal MI and nonfatal stroke), except for 4P-MACE for AMPLITUDE-O (3P-MACE outcomes plus death from undetermined causes)

**Table 2:** Current recommendations based on CVOTs for patients with established CVD or at high risk for CVD

Guidelines	Selected recommendations for CVD management based on diabetes CVOTs
ADA 2022	For patients with T2D who have established ASCVD or high / very high CV risk, SGLT2 inhibitors or GLP-1 RA with proven cardiovascular benefit are recommended as part of glycaemic management:* • Either a GLP-1 RA with proven CVD benefit or an SGLT2 inhibitor with proven CVD benefit • If further intensification is required or the patient is now unable to tolerate a GLP-1 RA and/or SGLT2 inhibitor choose agents demonstrating CV safety; consider adding the other class (GLP-1 RA or SGLT2 inhibitor) with proven CVD benefit^†^
ACC 2020	For patients with T2D who have established or high risk of ASCVD consider an SGLT2 inhibitor or GLP-1 RA with proven CV benefit
ADA and EASD 2019	For patients with T2D who have established ASCVD, an SGLT2 inhibitor or GLP-1 RA with proven cardiovascular benefit is recommended as part of glycaemic management: • First-line therapy is metformin • Add an GLP-1 RA with proven CVD benefit or, if eGFR is adequate, an SGLT2 inhibitor with proven CVD benefit • If further intensification is required or the patient is now unable to tolerate a GLP-1 RA and/or SGLT2 inhibitor, choose agents demonstrating CV safety^†^
ESC (in association with EASD) 2019	Consider CV risk independently of Hb1Ac; for patients with T2D who have ASCVD, or high/very high CV risk (target organ damage or multiple risk factors) • SGLT2 inhibitor or GLP-1 RA *(either as first add-on to metformin or as monotherapy; however, drug labels stipulate that metformin should be first line)* • If HbA1c is above target, consider adding the other class (GLP-1 RA or SGLT2i) with proven CVD benefit

A summary of recommendations in major international guidelines that are based on evidence from diabetes CVOTs. These guidelines include the American Diabetes Association (ADA) Standards of Medical Care in Diabetes 2022 [44]; American College of Cardiology (ACC) 2020 Expert Consensus Decision Pathway on Novel Therapies for Cardiovascular Risk Reduction in Patients with Type 2 Diabetes and Atherosclerotic Cardiovascular Disease [39]; Management of hyperglycaemia in type 2 diabetes, 2018: A consensus report by the ADA and the European Association for the Study of Diabetes (EASD), together with its 2019 update [40, 42]; 2019 European Society of Cardiology (ESC) Guidelines on diabetes, pre-diabetes, and cardiovascular diseases developed in collaboration with the EASD [38]

ASCVD, atherosclerotic cardiovascular disease; CV, cardiovascular; CVD, cardiovascular disease; CVOT, cardiovascular outcomes trial; GLP-1 RA, glucagon-like peptide-1 receptor agonist; Hb1Ac, haemoglobin A1c; SGLT2, sodium–glucose transporter 2

*Other options are thiazolidinediones, DPP-4 inhibitors if not on GLP RA, basal insulin, sulfonylureas

^†^Based on the flowchart of treatment of patients with T2D in the ADA 2022 guidelines, “first-line therapy depends on comorbidities, patient-centred treatment factors, including cost and access considerations, and management needs and generally includes metformin and comprehensive lifestyle modification”, and treatment choices are subsequently shown on the flowchart according to the presence/absence of ASCVD, indicators of high risk, heart failure, and chronic kidney disease

The purpose of this review is to provide an expert summary that will help clinicians navigate the overwhelming wealth of CVOT data. We discuss how CVOTs can provide valuable insights for management in clinical practice, and consider remaining gaps in knowledge, as well as how diabetes CVOTs have led to further cardiorenal-focussed studies that seek to understand more about how some GLDs may improve outcomes for our patients.

## Can we compare diabetes CVOTs?

In the absence of head-to-head studies, caution must be exercised when interpreting data from indirect comparison of CVOTs. Among the potential heterogeneity in trial designs and baseline characteristics, particular attention should be paid to differing baseline criteria for CVD diagnosis and CV risk in trial cohorts; patients with established CVD or CV risk factors at baseline may be more likely to progress through the continuum of CVD [45]. The proportions of patients with established CVD varied substantially between the CVOTs. For instance, all patients in ELIXA had established CVD, compared with 31–83% in LEADER, SUSTAIN-6 and REWIND (Additional file 2: Figure S1). Other key baseline characteristics that varied substantially between the CVOTs included HF diagnosis and renal impairment. There have also been suggestions of differing outcomes by region or race/ethnicity in the CVOTs, and in the HF and renal outcome trials, although these studies were not powered to reliably detect differences between subgroups [27, 30, 32, 46]. For instance, as recently reported for the LEADER CVOT of the GLP-1 RA liraglutide, 3P-MACE HR (95% CI) ranged from 0.62 (0.37–1.04) in Asia to 1.01 (0.84–1.22) in North America, although there was a lack of clear statistical evidence of interaction between regions and the outcome (p = 0.20) [32, 47]. The task of assessing the profile of CV risk in CVOT populations is also complicated by the prevalence of unrecognised diabetic cardiac impairment in patients with T2D, which may include ischaemia, myocardial dysfunction and/or cardiac arrhythmia presenting with atypical symptoms [48]. However, it is notable that post hoc analyses of EMPA-REG OUTCOME showed consistency of CV benefits with empagliflozin across patients with different baseline CV risk factors, including prior MI [49], prior stroke [49], Thrombolysis In Myocardial Infarction (TIMI) score [49], prior coronary artery bypass graft surgery [50], left ventricular hypertrophy [51], peripheral artery disease [52] and atrial fibrillation [53]. Canagliflozin has also shown consistency in CV outcomes across subgroups, including in patients with different levels of albuminuria [54], and enhanced 3P-MACE in patients with prior diuretic usage [55].

## From CV safety to CV efficacy in patients with T2D

### DPP-4 inhibitors: no evidence for cardioprotection

The first T2D CVOTs to be reported, SAVOR-TIMI 53 and EXAMINE, assessed the CV safety of the DPP-4 inhibitors saxagliptin and alogliptin, respectively. Before publication of these two CVOTs in 2013, post hoc analyses of phase 2 and 3 trials suggested a trend for lower incidence of major CV events with DPP-4 inhibitors than with placebo or other comparators [56]. Similarly, both CVOTs demonstrated non-inferiority in 3P-MACE for saxagliptin (HR [95% CI] 1.00 [0.89–1.12]) and alogliptin (HR [95% CI] 0.96 [upper < 1.16]), compared with placebo (Additional file 1: Table S1) [57, 58]. However, saxagliptin had a significantly elevated risk of HHF compared with placebo (HR [95% CI] 1.27 [1.07–1.51], p < 0.01) [57] and there was a suggestion of increased risk of HHF in patients treated with alogliptin vs placebo (HR [95% CI] 1.19 [0.90–1.58]), which led to the FDA issuing a safety warning for both alogliptin and saxagliptin [59]. Overall, subsequent CVOTs for DPP-4 inhibitors (sitagliptin and linagliptin) have demonstrated acceptable CV safety, consistently showing a neutral effect on 3P-MACE [13, 14, 60]. CARMELINA (linagliptin) included a cohort with a majority of patients presenting with prevalent chronic kidney disease (CKD) at baseline (mean estimated glomerular filtration rate [eGFR], 55 mL/min/1.73 m^2^) [20]. In the CAROLINA CVOT (mean eGFR at baseline, 77 mL/min/1.73 m^2^), linagliptin was non-inferior to glimepiride, based on 3P-MACE [21].

### SGLT2 inhibitors: cardioprotection with empagliflozin and canagliflozin

Cardioprotective benefits of GLDs were first observed in the EMPA-REG OUTCOME trial, in which the SGLT2 inhibitor empagliflozin showed a 14% reduction in the risk of 3P-MACE compared with placebo (HR [95% CI] 0.86 [0.74–0.99], p = 0.04) in patients with T2D and established CVD [27]. Among the components of 3P-MACE, the risk of CV death was reduced by 38% with empagliflozin (HR [95% CI] 0.62 [0.49–0.77], p < 0.001), while the impact on each of nonfatal stroke and nonfatal MI was neutral [27] (Table 1; Additional file 1: Table S1).

The canagliflozin CVOT programme, comprising CANVAS and CANVAS-R, also demonstrated a 14% reduction in 3P-MACE (HR [95% CI] 0.86 [0.75–0.97], p = 0.02) in patients with established CVD or high CV risk, although no significant reduction in CV deaths (HR [95% CI] 0.87 [0.72–1.06]) [30]. The beneficial effect of canagliflozin on 3P-MACE was confirmed in patients with T2D and CKD in a subsequent renal outcomes trial, CREDENCE (HR [95% CI] 0.80 [0.67–0.95], p = 0.01), which also showed a trend towards a reduction in CV deaths that neared significance (HR [95% CI] 0.78 [0.61–1.00], p = 0.05) [36]. CKD in patients with T2D has been strongly linked to CV events and mortality in CVOTs [14], although the prevalence of CKD in diabetes CVOTs was typically much lower than in CREDENCE [14, 36].

A recently reported meta-analysis of 11 clinical trials demonstrated cardiorenal benefits across the SGLT2 inhibitor class versus placebo. CV benefits included a 12% reduction in 3P-MACE (without significant heterogeneity; I^2^ = 21.2%, p = 0.19), based on six cardiorenal studies that reported this outcome, and a 16% reduction in CV death [61]. However, these results should be caveated; there were differences in outcomes, study designs, patient populations, and medications across the cardiorenal studies included in the meta-analysis. The 12% reduction in 3P-MACE was based on data from EMPA-REG OUTCOME, CANVAS, CREDENCE, DECLARE-TIMI 58 (dapagliflozin), VERTIS CV (ertugliflozin) and SCORED (sotagliflozin). Notably, sotagliflozin has both SGLT1 and SGLT2 inhibitory activity and is not a licensed treatment for T2D (but is licensed for type 1 diabetes in Europe), and SCORED was a cardiorenal study (patients had T2D and CKD) that used a different 3P-MACE outcome (CV death, HHF and urgent visits for HF) than the other studies (CV death, nonfatal MI and nonfatal stroke). The dapagliflozin CVOT, DECLARE-TIMI 58, did not show a benefit in either 3P-MACE (HR [95% CI] 0.93 [0.84–1.03], p = 0.17) or CV deaths (0.98 [0.82–1.17]) [37, 62]. However, DECLARE-TIMI 58 had a very different profile of baseline characteristics to EMPA-REG OUTCOME and CANVAS, as a majority of patients had high CV risk but not established CVD, and there were fewer patients with CKD [37]. Therefore, the different outcomes in DECLARE-TIMI 58, compared with EMPA-REG OUTCOME and CANVAS, may be due to differences in study design and cohort composition rather than intrinsic differences between the study drugs. Two HF and renal outcomes studies, designed to assess the effect of dapagliflozin vs placebo in patients with HF with reduced ejection fraction (HFrEF; DAPA-HF) or CKD (DAPA-CKD) with or without T2D, both reported trends towards reductions in CV death in the T2D subgroups (HR [95% CI] 0.79 [0.63–1.01] and 0.85 [0.59–1.21], respectively) [63, 64]. In the VERTIS CV study of ertugliflozin, all patients had established CVD at baseline, but no benefit was observed in 3P-MACE (HR [95% CI] 0.97 [0.85–1.11]) or CV death (HR [95% CI] 0.92 [0.77–1.11]) [16]. These findings suggest that significant improvements in CV outcomes, which were observed in CVOTs of empagliflozin and canagliflozin, may not apply to all SGLT2 inhibitors.

### GLP-1 RAs: cardioprotection with subcutaneous and long acting GLP-1 RAs, but inconclusive evidence for short-acting and oral long-acting medications

A meta-analysis of eight CVOTs recently demonstrated reductions in 3P/4P-MACE and CV death of 14% and 13%, respectively, across the GLP-1 RA class, compared with placebo [65]. These findings were based on data from five studies of subcutaneously administered long-acting GLP-1 RAs (AMPLITUDE-O, LEADER, SUSTAIN-6, REWIND, and HARMONY OUTCOMES), a study of orally administered long-acting semaglutide (PIONEER-6) and two studies of subcutaneously administered short-acting GLP-1 RAs (ELIXA, EXSCEL). The FREEDOM-CVO non-inferiority study of continuously infused exenatide, which recently showed no CV benefits over placebo based on the primary outcome of 4P-MACE (HR [95% CI] 1.21 [0.90–1.63]), 3P-MACE and their individual component outcomes [24] (Additional file 1: Table S1), was not included in the meta-analysis.

Significant reductions in 3P/4P-MACE have been reported for all five of the CVOTs of subcutaneously administered long-acting GLP-1 RAs, including the recently reported AMPLITUDE-O study (efpeglenatide; HR [95% CI] 0.73 [0.58–0.92]; p < 0.01), LEADER (liraglutide; 0.87 [0.78–0.97], p = 0.01), SUSTAIN-6 (semaglutide; 0.74 [0.58–0.95], p = 0.02), REWIND (dulaglutide; 0.88 [0.79–0.99], p = 0.03), and HARMONY OUTCOMES (albiglutide; 0.78 [0.68–0.90], p < 0.01) (Table 1) [31, 32, 34, 35]. The latter GLP-1 RA, albiglutide, is no longer commercially available.

When the oral formulation of semaglutide was compared with placebo in the PIONEER-6 trial [15], a trend was observed towards reduction in 3P-MACE (HR [95% CI] 0.79 [0.57–1.11], p = 0.17). However, PIONEER-6 was a small study (N = 3183) of short duration, designed to rule out excess risk of 3P-MACE, and not powered to demonstrate superiority [15]. Based on clinicaltrials.gov, a large CVOT investigating an oral formulation of semaglutide, the SOUL trial, is underway (estimated N = 9642). Primary and study completion are scheduled for July 2024.

Across the long-acting GLP-1 RA CVOTs, the outcomes for individual components of 3P-MACE were much less uniform than for the composite endpoint: only two of the five trials demonstrated a significant reduction in CV death, LEADER (liraglutide; HR [95% CI] 0.78 [0.66–0.93], p = 0.01) and PIONEER-6 (oral semaglutide; HR [95% CI] 0.49 [0.27–0.92], p = 0.03) [15, 32, 66]; however, neither study showed a significant reduction in nonfatal stroke or nonfatal MI, whereas SUSTAIN-6 (semaglutide) and REWIND (dulaglutide) significantly reduced the risk of nonfatal stroke, while HARMONY OUTCOMES (albiglutide) significantly reduced the risk of fatal or nonfatal MI [31, 34, 35].

Unlike the findings for long-acting GLP-1 RAs, the short-acting GLP-1 RA lixisenatide showed no significant CV benefits in the ELIXA study, taking into account 4P-MACE (HR [95% CI] 1.02 [0.89–1.17]; p = 0.81), its individual components, and HHF [26] (Additional file 1: Table S1). The EXSCEL study of prolonged-release exenatide, another short-acting GLP-1 RA, showed a trend towards a reduction in 3P-MACE that neared significance (HR [95% CI] 0.91 [0.83–1.00], p = 0.06) [33] although, as previously mentioned, no CV benefits were observed for continuously infused exenatide in the FREEDOM-CVO trial [24]. In addition to the possibility of patients’ baseline characteristics affecting study outcomes, the differing results of the long- and short-acting GLP-1 RA CVOTs suggest that the kinetics of both receptor agonism and drug exposure may play roles in conferring cardioprotection. More research is needed to determine whether the documented differences between the pharmacokinetics, delivery and effects of short- and long-acting GLP-1 RAs [67] translate into differences in CV outcomes.

## Can modern glucose-lowering drugs reduce all-cause mortality?

The data emerging from CVOTs means that clinicians can, for the first time, consider therapeutic options among GLDs that may reduce mortality and improve CV outcomes in certain patient groups. Unlike DPP-4 inhibitors, SGLT2 inhibitors and some GLP-1 RAs are associated with significant reductions in all-cause mortality (Table 1 and Additional file 1: Table S1).

### SGLT2 inhibitors: evidence for reduced all-cause mortality

No significant reduction of all-cause death with dapagliflozin was seen in DECLARE-TIMI 58 (HR [95% CI] 0.93 [0.82–1.04]) [37]. However, reductions in all-cause death were observed in DAPA-HF (HR [95% CI] 0.83 [0.71–0.97]) and in DAPA-CKD (0.69 [0.53–0.88]), in populations of patients with HFrEF or CKD, with or without T2D. These reductions in all-cause death were compatible with CV death outcomes in DAPA-HF (HR [95% CI] 0.82 [0.69–0.98]) and in DAPA-CKD (0.81 [0.58–1.12]) [68, 69].

Notably, EMPA-REG OUTCOME (empagliflozin) demonstrated a significantly reduced all-cause death rate (HR [95% CI] 0.68 [0.57–0.82]) (Additional file 1: Table S1), which was primarily driven by a reduced risk of CV death (Table 1) [27, 32]. Another study, EMPEROR-Reduced, was designed to assess the effect of empagliflozin vs placebo in patients with HFrEF, with or without T2D. In this patient population, trends towards reductions in CV death were reported in patients with T2D (HR [95% CI] 0.92 [0.71–1.20]) and without T2D (0.92 [0.68–1.24]) [70].

In the canagliflozin diabetes CVOT programme (CANVAS and CANVAS-R), no statistically significant reductions were detected in all-cause mortality (HR [95% CI] 0.87 [0.74–1.01]) or CV deaths (0.87 [0.72–1.06]) in patients with T2D [30].

### GLP-1 RAs: evidence for reduced all-cause mortality

The LEADER CVOT demonstrated significantly reduced all-cause mortality with liraglutide vs placebo (HR [95% CI] 0.85 [0.74–0.97]) (Additional file 1: Table S1), compatible with reduced risk of CV death (Table 1) [27, 32]. A reduced risk of all-cause death in patients with T2D was also noted in EXSCEL (exenatide) (HR [95% CI] 0.86 [0.77–0.97]) and PIONEER-6 (oral semaglutide) (0.51 [0.31–0.84]), although these results were only nominally significant, owing to the hierarchical testing plans used [15, 33]. These reductions in all-cause death were accompanied by a trend towards reduction in CV death in EXSCEL (HR [95% CI] 0.88 [0.76–1.02]) and, as previously mentioned, by significant reduction in PIONEER-6 (0.49 [0.27–0.92], p = 0.03) (Additional file 1: Table S1).

Similarly, in the recently published AMPLITUDE-O CVOT (efpeglenatide), the trend towards reduction in CV death (HR [95% CI] 0.72 [0.50–1.03]) was compatible with all-cause mortality (0.78 [0.58–1.06]).

## Treatment recommendations in relation to CV benefits and reduced all-cause mortality

In light of the significant benefits of certain SGLT2 inhibitors and GLP-1 RAs in reducing the risks of CV death and all-cause death in patients with T2D, major international guidelines have been updated to include evidence from CVOTs to help differentiate between the use of GLDs. The American College of Cardiology (ACC) [39], American Diabetes Association (ADA) and European Association for the Study of Diabetes (EASD) [42, 44], and the Europe Society of Cardiology (ESC) and EASD [38] guidelines all recommend specific treatments for patients with T2D and atherosclerotic CVD (ASCVD) based on CVOT data (Table 2). The general consensus between the guidelines is that patients diagnosed with T2D and CVD should be treated with an SGLT2 inhibitor or GLP-1 RA with proven CVD benefit, either as first add-on to metformin or as monotherapy. The ESC guidelines specifically recommend use of empagliflozin in patients with T2D and CVD to reduce the risk of death, while empagliflozin, canagliflozin, or dapagliflozin are recommended in patients with T2D and CVD, or at very high/high CV risk, to reduce CV events [43]. Regarding choice of GLP-1 RA, the ESC and ACC guidelines recommend the use of dulaglutide, liraglutide or injectable semaglutide for patients with T2D and CVD, based on their CV benefits [38–40, 43].

## Beyond MACE: HF and renal findings

Many CVOTs have reported beyond the mandated 3P-MACE outcomes, elucidating additional benefits seen with some GLDs, including reducing the risk of HHF and slowing the progression of renal disease. For the most part, these have been secondary outcomes, although complementary dedicated HF and renal outcomes studies that included patients with and without T2D have recently been published for SGLT2 inhibitors [18, 36, 68, 69, 71, 72], while large-scale real-world outcomes studies have provided further insights [73–82].

### SGLT2 inhibitors: evidence for reduced risk of HHF

**Fig. 2:**
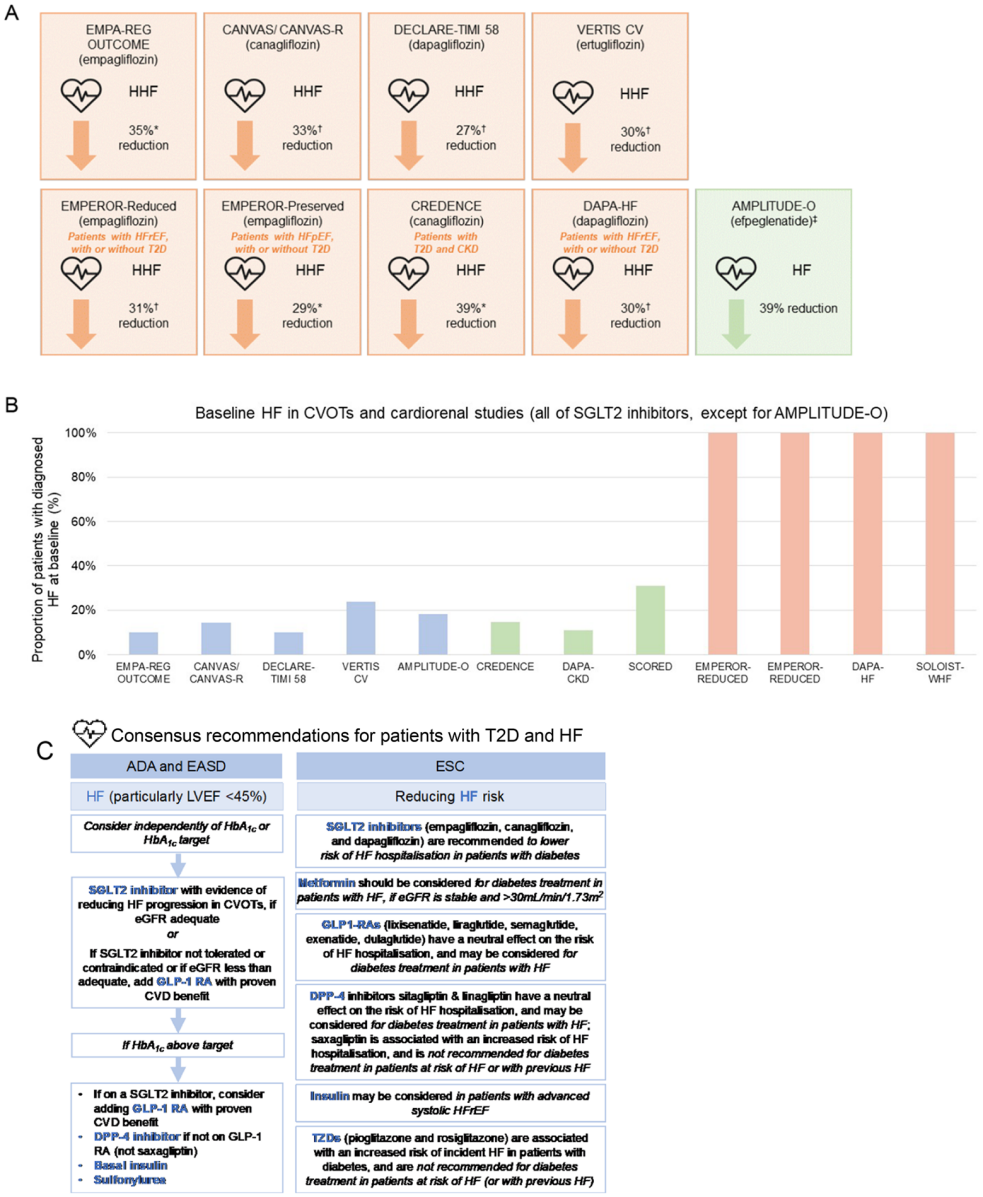

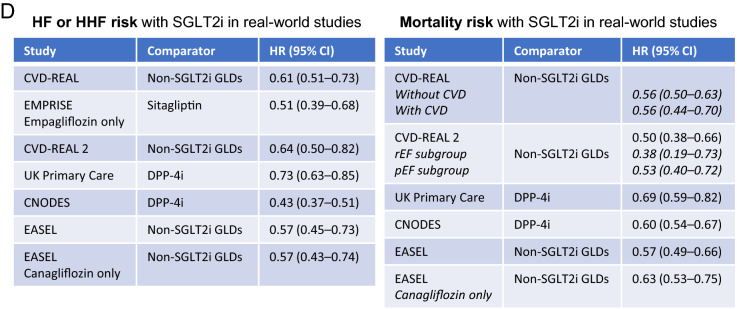
Exploring possible HF benefits with glucose-lowering drugs (mainly SGLT2 inhibitors). All clinical trials shown are of SLGT2 inhibitors, except for AMPLITUDE-O (efpeglenatide, GLP-1 RA). A consistent pattern of fewer HHF events, with a large effect size, has been seen across the SGLT2 inhibitor class [13, 62, 139]. These reductions were closely mirrored in a dedicated renal outcomes study of canagliflozin in patients with diabetic kidney disease [36], and in dedicated HF outcomes studies of dapagliflozin and empagliflozin in patients with HFrEF with or without diabetes [69, 71] and of empagliflozin in patients with HFpEF with or without diabetes [86] (**A**). Results from these trials are shown to illustrate the consistency of findings regarding HHF; they should not be directly compared, due to differences in study design, definitions and populations. Note that HHF as a standalone endpoint was not a primary outcome measure in any of the studies shown and has not been reported for the SOLOIST-WHF HF outcomes study, DAPA-CKD renal outcomes or SCORED cardiorenal studies. Diabetes CVOTs were not initially designed to assess any protective effect on HHF (for example, most patients were not diagnosed with HF at baseline (**B**) [16, 18, 27, 30, 36, 37, 63, 69, 70, 72]). International guidelines for the treatment of patients with T2D now recommend SGLT2 inhibitors to protect patients from HF [38, 40, 42] (**C**), while real-world studies have confirmed the pattern of fewer HHF events in the more diverse patients seen in routine clinical practice [73, 74, 77–82, 89, 90] (**D**). ACC, American College of Cardiology; ADA, American Diabetes Association; CI, confidence interval; CVD, cardiovascular disease; CVOT, cardiovascular outcomes trial; DPP-4, dipeptidyl peptidase-4; EASD, European Association for the Study of Diabetes; ESC, European Society of Cardiology; GLD, glucose lowering drug; GLP-1 RA, glucagon-like peptide-1 receptor agonist; Hb1Ac, haemoglobin A1c; HF, heart failure; HFpEF, HF with preserved ejection fraction; HFrEF, HF with reduced ejection fraction; HHF, hospitalisation for HF; HR, hazard ratio; SGLT2, sodium–glucose transporter 2; T2D, type 2 diabetes; TZD, thiazolidinedione. *p < 0.05. ^†^Exploratory analysis. ^‡^Efpeglenatide is not a currently licensed treatment

Both dapagliflozin and empagliflozin are approved in Europe and the US for the treatment of patients with chronic HFrEF, based on published findings of dedicated HF outcomes studies, DAPA-HF and EMPEROR-Reduced (Fig. 2A, B) [69, 71, 83, 84]. In February and March 2022, empagliflozin also received FDA and European Commission approval for the treatment of patients with preserved EF (HFpEF), in light of encouraging findings from the recently reported EMPEROR-Preserved trial [85, 86], while the DELIVER trial of dapagliflozin in patients with HFpEF is ongoing [87]. The recently completed EMPEROR-Preserved and ongoing DELIVER trials are covered in the ‘Where Next?’ section of this review.

In the DAPA-HF cohort of patients with HFrEF, only 40% of which had comorbid T2D, the relative risk reduction (RRR) for HHF was 30% with dapagliflozin in the overall population (Additional file 1: Table S1) [69]; when looking only at patients with T2D, the RRR observed was 24% [64]. Very similar results were seen with empagliflozin in patients with HFrEF in EMPEROR-Reduced, with RRR of 31% in HHF for all patients and 33% when only looking at those with T2D [70, 71]. The results of a recent meta-analysis of patients with HFrEF from DAPA-HF and EMPEROR-Reduced demonstrated consistent CV benefits, based on a composite of HHF and CV death, for a range of patient subgroups including those with or without T2D and regardless of baseline eGFR (i.e. above or below 60 mL/min/1.73 m^3^) [88]. The protection from HHF offered by SGLT2 inhibitors has now been reflected in international guidelines [38, 40, 42] and in several real-world studies (Fig. 2D) [73, 74, 77–80, 89, 90].

In addition to dedicated HF outcomes studies, a reduced risk of HHF in patients with T2D has also been demonstrated consistently in diabetes CVOTs and in renal outcomes studies across a range of SGLT2 inhibitors, including empagliflozin (EMPA-REG OUTCOME, RRR 35%) [27], canagliflozin (CANVAS/CANVAS-R, RRR 33%; CREDENCE, RRR 39%) [30, 36], dapagliflozin (DECLARE-TIMI 58, RRR 27%) [37] and ertugliflozin (VERTIS CV, RRR 30%) (Fig. 2A) [16]. Indirect comparison of these findings is hampered by baseline HF not being well characterised in the CVOT patient cohorts, by variation in baseline characteristics between studies, and by lack of power to detect an impact on HHF. For instance, across the SGLT2 inhibitor CVOTs, the proportion of patients with HF diagnosed at baseline ranged from 10% in EMPA-REG OUTCOME to 24% in VERTIS CV (Fig. 2B; Additional file 1: Table S1) [16, 27, 30, 36, 37]. Nevertheless, these shortcomings have been at least partly overcome by dedicated HF outcomes trials.

### SGLT2 inhibitors: evidence for renal benefits

**Fig. 3:**
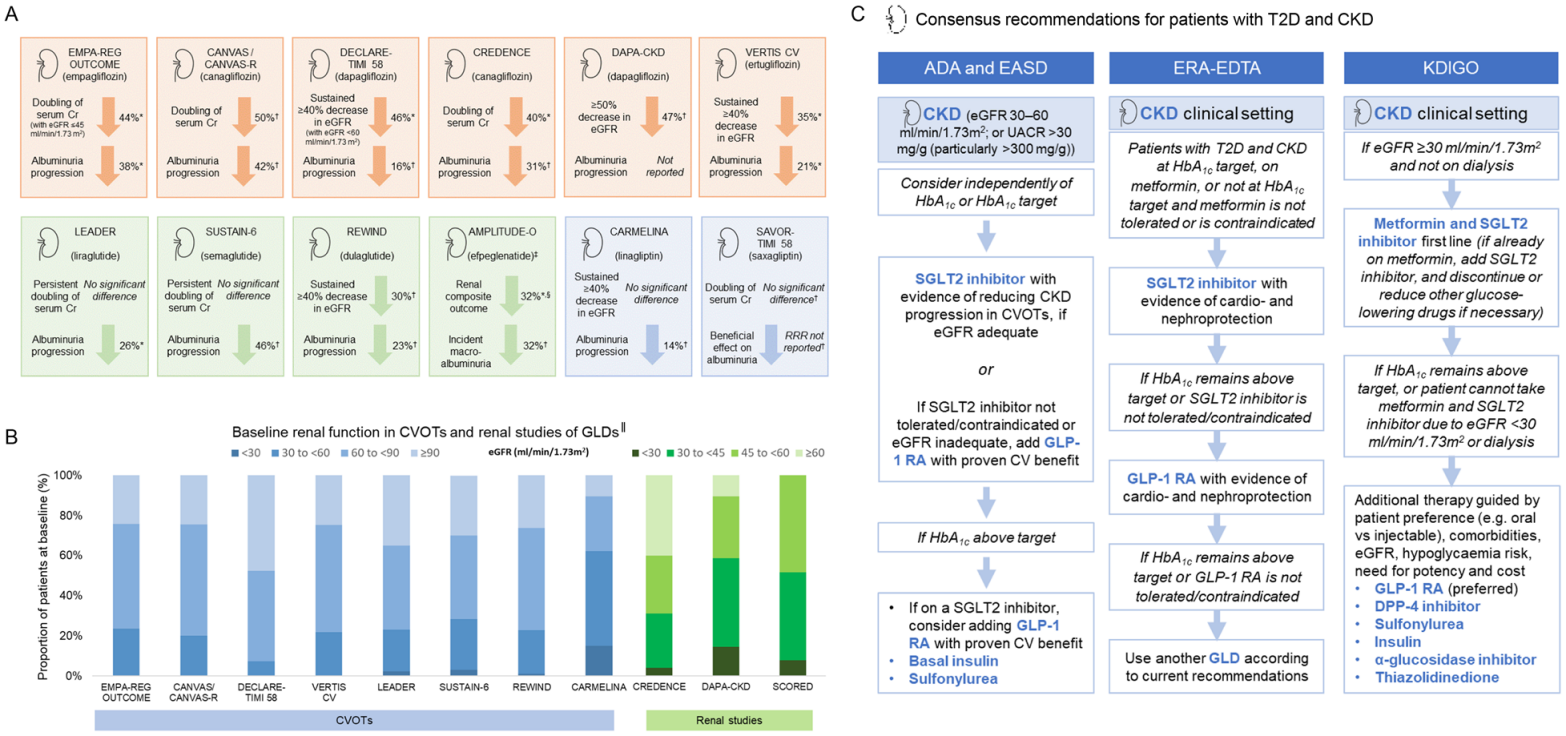
Exploring possible renal benefits with glucose-lowering drugs. CVOTs typically included renal endpoints among secondary outcomes. Effects on renal outcomes have been generally consistent between studies—showing a reduced risk for progression of renal impairment with SGLT2 inhibitors [28, 36, 37, 92, 95, 140], and a slowed progression of albuminuria with both SGLT2 inhibitors [28, 30, 36, 95] and GLP-1 RAs [31, 96, 97]. In addition to CVOTs, renal benefits of SGLT2 inhibitors have been shown in dedicated renal outcomes trials (CREDENCE and DAPA-CKD) [20, 36, 68, 72] (**A**). Dulaglutide showed a benefit for some renal impairment outcomes in an exploratory analysis of the REWIND CVOT [97]. Renal outcomes with DPP-4 inhibitors in CVOTs have typically been neutral, although linagliptin showed a modest benefit regarding reduced progression of albuminuria in CARMELINA, a CVOT notable for the prevalence of CKD among the population [14], and SAVOR-TIMI 53 showed a slower progression of albuminuria with saxagliptin compared with placebo [100]. Note that trials differed significantly in the measures used to assess renal function and albuminuria progression, and there was also a large variation in renal risk at baseline (for example, degree of renal impairment [20, 27, 31, 36, 63, 72, 96, 97, 125, 126, 140–142]), and therefore should not be directly compared [14] (**B**). While we await further results from dedicated renal studies, the consistency of effect size in slowing renal function decline has been sufficiently persuasive to lead to updated guidelines recommending SGLT2 inhibitors for patients with T2D in a CKD setting [40–42, 44, 102] (**C**). ADA, American Diabetes Association; CKD, chronic kidney disease; Cr, creatinine; CVD, cardiovascular disease; CVOT, cardiovascular outcomes trial; EASD, European Association for the Study of Diabetes; EDTA–ERA, European Dialysis and Transplant Association–European Renal Association; eGFR, estimated glomerular filtration rate; ESRD, end-stage renal disease; GLD, glucose lowering drug; GLP-1 RA, glucagon-like peptide-1 receptor agonist; Hb1Ac, haemoglobin A1c; KDIGO, Kidney Disease Improving Global Outcomes; RRT, renal-replacement therapy; RRR, relative risk reduction; SGLT2, sodium–glucose transporter 2; UACR, urinary albumin-to-creatinine ratio. *p < 0.05. ^†^Exploratory analysis. ^‡^Efpeglenatide is not a currently licensed treatment. ^§^In AMPLITUDE-O, the composite renal outcome was incident macroalbuminuria (UACR > 300 mg/g or > 33.9 mg/mmol), ≥ 30% increase in UACR from baseline, sustained ≥ 40% decrease in eGFR for ≥ 30 days, renal-replacement therapy for ≥ 90 days, and sustained eGFR of < 15 mL/min/1.73 m^2^ for ≥ 30 days). ^‖^In AMPLITUDE-O, 31.6% of patients had eGFR < 60 mL/min/1.73 m^2^, and proportions of patients with other eGFR levels were not reported; eGFR < 25 mL/min/1.73 m^2^ was an exclusion criterion

Dapagliflozin recently became the first SGLT2 inhibitor approved in Europe for the treatment of patients with CKD, regardless of diabetes status, based on findings from the DAPA-CKD renal outcomes trial. Adding dapagliflozin to standard care was associated with significantly lower risk (HR [95% CI] 0.61 [0.51–0.72], p < 0.001) of a composite cardiorenal outcome (sustained decline in the eGFR of ≥ 50%, end-stage kidney disease, or death from renal or CV causes) and other renal benefits (Fig. 3A) [68, 91]. Another dedicated renal outcomes study (CREDENCE), in patients with T2D and comorbid CKD, also confirmed the profile of renal benefits with canagliflozin suggested by the CANVAS diabetes CVOT programme (Fig. 3A) [36]. Improved renal outcomes have been noted consistently across CVOTs for SGLT2 inhibitors, both in terms of renal function and albuminuria. RRR in renal function outcomes were ≥ 35% across the class (Fig. 3A) [28, 36, 37, 92–94]. Progression of albuminuria was also consistently slowed with SGLT2 inhibitors (Fig. 3A) [28, 30, 36, 93, 95]. In the SCORED cardiorenal study (sotagliflozin), there was a trend towards benefit (HR [95% CI] 0.71 [0.46–1.08]) for a composite of renal outcomes (first occurrence of a sustained decrease of ≥ 50% in eGFR from baseline for ≥ 30 days, long-term dialysis, renal transplantation, or sustained eGFR of < 15 mL/min/1.73 m^2^ for ≥ 30 days) in patients with T2D and comorbid CKD [72]. Note that sotagliflozin is not a licensed treatment for T2D and has both SGLT1 and SGLT2 inhibitory activity.

### GLP-1 RAs: potential reduction in HHF and evidence for some renal benefits

While GLP-1 RA CVOTs demonstrated improvements in some renal outcomes relating to albuminuria, neutral effects were typically seen on the hard endpoint of renal function (Fig. 3A) and, when reported, on HHF (Additional file 1: Table S1) [23, 31, 34, 96]. However, the recently published AMPLITUDE-O CVOT demonstrated RRRs of 39% for HF, 32% for incident macroalbuminuria, and 32% for a composite renal outcome (incident macroalbuminuria, ≥ 30% increase in UACR from baseline, sustained ≥ 40% decrease in eGFR for ≥ 30 days, renal-replacement therapy for ≥ 90 days, and sustained eGFR of < 15 mL/min/1.73 m^2^ for ≥ 30 days) with efpeglenatide vs placebo [23]. A trend towards a decrease with efpeglenatide (HR [95% CI] 0.77 [0.57–1.02], p = 0.07) was reported for another renal composite outcome (≥ 40% decrease in eGFR for ≥ 30 days, end-stage kidney disease, or death from any cause) [23]. REWIND (dulaglutide) also showed benefits for some, but not all, measures of kidney function [97].

### DPP-4 inhibitors: neutral effect on HHF, in general, and evidence for modest renal benefits

CVOTs investigating DPP-4 inhibitors have generally shown neutral effects on HHF and modest renal benefits in terms of reduced albuminuria [20, 98–100]. In CARMELINA, linagliptin demonstrated a modest reduction in time to first occurrence of albuminuria progression vs placebo (RRR 14%) (Fig. 3A) [20]. In SAVOR-TIMI 53, saxagliptin showed beneficial albuminuria results (RRR not reported) [100] but also an elevation in HHF [57], while EXAMINE (alogliptin) reported a trend towards increased HHF [59].

## Treatment recommendations in relation to HF and renal benefits

The prevalence of renal impairment across diabetes CVOTs varied considerably, being particularly high in CARMELINA (linagliptin), hampering conclusions about how renal effects may compare between GLDs (Fig. 3B) [14, 20, 101]. However, the totality of evidence from CVOTs and renal outcomes studies shows conclusively that patients with T2D experience superior renal benefits with SGLT2 inhibitors than with DPP-4 inhibitors and currently approved GLP-1 RAs.

Moreover, despite the limitations of CVOTs for assessing HF and renal outcomes, the evidence for HF and renal benefits with SGLT2 inhibitors was deemed sufficient by professional societies to update guidelines, even before the emergence of results from dedicated HF and renal studies. As such, SGLT2 inhibitors are recommended as either first add-on, concomitant to metformin, or as a monotherapy in patients with T2D and HF or CKD in guidelines that include the ADA and EASD joint Consensus Report on the Management of Hyperglycaemia 2019 [42], the ADA’s Standards of Medical Care in Diabetes 2022 [44], the European Renal Association (ERA)—European Dialysis and Transplant Association (EDTA) 2019 guidelines [102], and the Kidney Disease Improving Global Outcomes (KDIGO) 2020 guidelines on diabetes management in CKD [41] (Figs. 2C, 3C).

## Other clinical considerations

In addition to considering the impact of GLDs on cardiorenal outcomes from CVOTs and related studies, there are also other practical reasons to prescribe DPP-4 inhibitors, GLP-1 RAs, and SGLT2 inhibitors. For instance, all three therapeutic classes are associated with relatively low risk of hypoglycaemic events, while patients treated GLP-1 RAs and SGLT2 inhibitors may benefit from weight loss [15, 27, 30, 32, 57, 103].

## Clinical inertia to the use of SGLT2 inhibitors and GLP-1 RAs

Many patients with CV risk still do not receive SGLT2 inhibitors or GLP-1 RAs as part of their GLD regimen, even though these medications are recommended for CVD prevention in the treatment guidelines. DPP-4 inhibitors are more widely used than SGLT2 inhibitors or GLP-1 RAs, despite comparable costs to SGLT2 inhibitors and the lack of evidence that DPP-4 inhibitors improve cardiorenal outcomes [104]. The successful implementation of CVOT insights and new guidelines into clinical practice, and consequent improvements in patient outcomes, will rely heavily on implementation programmes and educational tools [38, 105].

## Where next?

Despite significant advancements in the treatment strategies available to patients with T2D (and endorsement in updated guidelines), outstanding questions are being addressed by ongoing research. Given that some CVOTs have populations entirely (or almost entirely) comprised of patients with established CVD, while other CVOTs also included patients at high risk of CVD events, greater insight into cardiorenal outcomes in these respective patient groups would be beneficial. Additional efficacy data for other patient subgroups would also be welcome, including investigation of potential differences in CV outcomes by region/ethnicity [47], and further investigation of GLDs in populations without T2D. Questions also remain regarding cost-effectiveness in particular patient subgroups, although SGLT2 inhibitors, GLP-1 RAs and DPP-4 inhibitors are generally considered to be cost-effective compared with insulin, thiazolidinediones and sulfonylureas in patients with T2D [106, 107].

### Combination therapy

Another avenue being explored is the potential value of combining different classes of GLD therapies; SGLT2 inhibition combined with GLP-1 RAs may have synergistic effects on HbA1c level, blood pressure, body weight, and CV outcomes [108, 109]. Regarding combination therapy with metformin, results from the GRADE randomised trial were presented at the EASD 2021 annual meeting; patients (N = 5047) received either glimepiride (sulfonylurea), sitagliptin (DPP-4 inhibitor), liraglutide (GLP-1 RA), or insulin glargine (clinicaltrials.gov identifier: NCT01794143). Incidence of CVD (MACE, HHF, unstable angina, revascularisation) was lowest with liraglutide, while microvascular (kidney and neuropathy) outcomes were comparable across the four treatment groups. The worst metabolic outcomes were observed with the combination of sitagliptin and metformin; the sitagliptin and glimepiride groups both met the primary outcome (≥ 7% HbA1c) more frequently, and earlier in time, than the glargine and liraglutide groups. Conversely, it is worth noting that linagliptin, another DPP-4 inhibitor, was significantly better than glimepiride regarding two key metabolic outcomes in the CAROLINA CVOT (both were composite outcomes that included maintenance of HbA1c at ≤ 7.0%, without > 2% weight gain) [21].

### Elucidating mechanisms of action in relation to cardiorenal protection

**Fig. 4:**
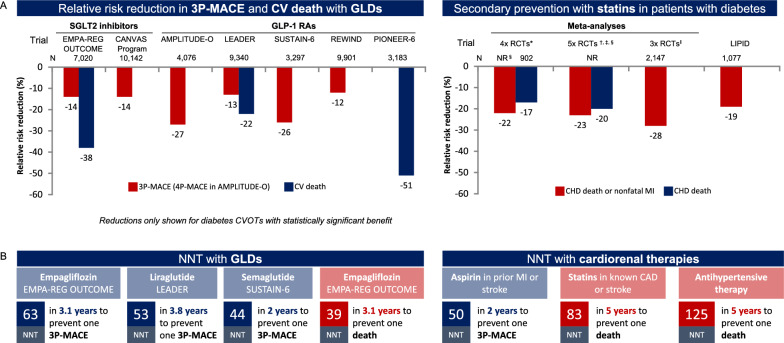
Diabetes CVOTs in the broader context of cardiology trials. PIONEER-6 was a small study (N = 3183) of short duration, designed to rule out excess risk of 3P-MACE, and not powered to demonstrate superiority. Certain diabetes CVOTs have shown cardiorenal protective effects that may arguably be comparable to outcomes with cardiorenal therapies [116], such as the relative risk reduction of CV events compared with statins [27, 30–32, 34, 143, 144] (**A**), or NNT to prevent CV events compared with statins, aspirin or antihypertensive therapy [27, 116, 145, 146] (**B**). For example, patients with diabetes and CVD in the LIPID trial had a 19% reduced risk of CHD death or nonfatal MI over 6 years with the statin pravastatin compared with placebo [143]; meta-analyses of secondary prevention in patients with diabetes in multiple statin trials have produced similar results [143, 144, 147]. 3P-MACE, 3-point major adverse CV event; CHD, coronary heart disease; CV, cardiovascular; CVOT, CV outcomes trial; GLD, glucose-lowering drug; GLP-1 RA, glucagon-like peptide-1 receptor agonist; MI, myocardial infarction; NNT, number needed to treat; NR, not reported; RCT, randomised control trial; SGLT2, sodium–glucose transporter 2. *Four RCTs (4S, CARE, Post-CABG and VA-HIT) for CHD death and nonfatal MI, and 3 RCTs (4S, CARE, Post-CABG) for CHD death. ^†^Five RCTs (4S, CARE, LIPID, Post-CABG and VA-HIT). ^‡^Includes 1 RCT that investigated a non-statin cholesterol-lowering drug. ^‖^Three RCTs (4S, CARE, LIPID)

Questions remain about the mechanism of action of SGLT2 inhibitors and GLP-RAs, particularly in relation to the cardiorenal benefits observed in some diabetes CVOTs [110–112] (Additional file 2: Fig. S2). Cardio- and reno-protective effects are unlikely to be solely explained by the mechanisms used by these drugs to lower blood glucose levels, as the same effects are not seen with drugs that have stronger antihyperglycaemic actions [111], and were not dependent upon the degree of HbA1c reduction [113–115]. Moreover, while direct comparisons cannot be made without head-to-head trials, some outcomes in diabetes CVOTs have been within the range expected for cardiorenal therapies such as statins, aspirin and antihypertensives [116], despite being added on top of a standard of care that often included these therapies (Fig. 4). Consequently, new theories around the modes of action for SGLT2 inhibitors and GLP-1 RAs are being hypothesised [112, 117–119], although as yet it remains unclear which mechanism(s) are responsible, or whether there is any mechanistic overlap between cardiorenal benefits with SGLT2 inhibitors and GLP-1 RAs.

Proposed, sometimes contradictory, mechanisms for cardiorenal protective effects with SGLT2 inhibitors include enhancement of fuel supply through the production of ketones (the “thrifty substrate” hypothesis) [118, 119]; an induction of tissue-protective, energy-preserving metabolic states similar to those seen with animal hibernation [112, 117]; haemodynamic volume effects (SGLT2 inhibitors are predicted to produce a twofold greater reduction in interstitial fluid volume compared with blood volume) [120]; improved cardiac remodelling, increased provascular progenitor cells and decreased ischaemia/reperfusion injury [121]; off-target inhibition of the cardiac Na^+^/H^+^ exchanger, thus reducing cardiac cytosolic sodium in animal models [122]; and possible direct influences of SGLT2 inhibitors on inflammatory responses [123]. More exhaustive lists of speculated mechanisms have been reviewed elsewhere [121].

For GLP-1 RAs, proposed mechanisms of action include an anti-atherothrombotic effect, as well as amelioration of inflammatory markers, resulting in the enhanced retardation of atherosclerosis [60, 124].

### Recently completed and ongoing HF and renal studies

**Fig. 5:**
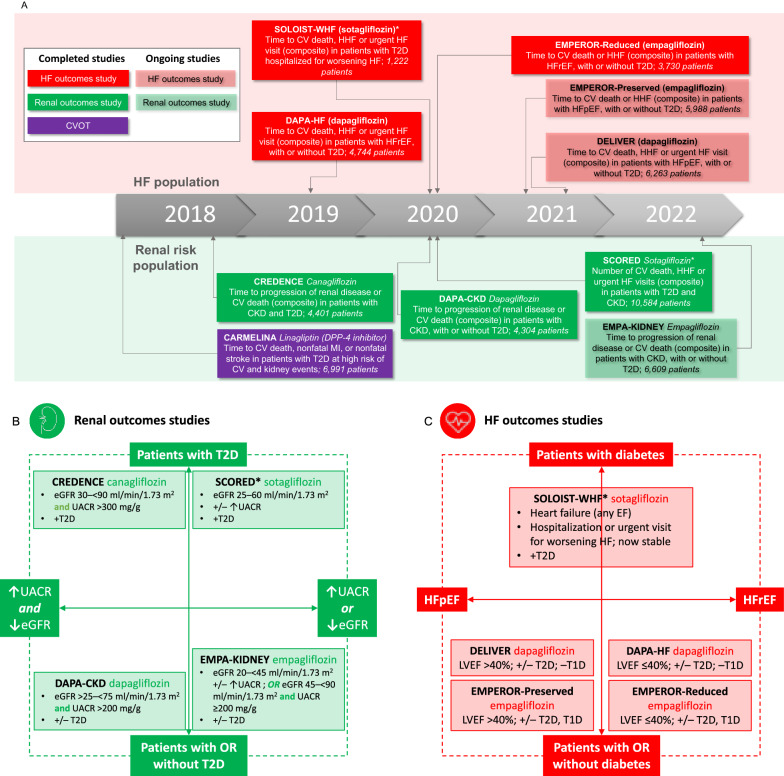
Completed and ongoing studies of SGLT2 inhibitors (and linagliptin) in renal risk or HF populations. Secondary HF and renal outcome measures in diabetes CVOTs of SGLT2 inhibitors were hypothesis generating, suggesting possible protective events on HF and renal disease. Only one diabetes CVOT (CARMELINA) included a majority of patients with CKD [101] (**A**); however, this was a study not on an SGLT2 but on a DPP-4 inhibitor, linagliptin, and was designed to demonstrate CV safety in a renal risk population, and not renal protection [101]. Subsequently, several dedicated HF [18, 69, 71, 148, 149] and renal [36, 68, 72, 150] outcome studies have been completed, or are underway, including studies that include patients with HF (**B**) or CKD (**C**) without diabetes [63, 68, 69, 71, 86, 148, 149, 151]. Among HF studies, both HFrEF [18, 69, 71] and HFpEF [18, 86, 148, 149] have recently or are being investigated (**B**), while renal studies include populations with albuminuria and/or with impaired renal function [36, 68, 72, 150] (**C**). −, without; +, with; +/−, with or without; CKD, chronic kidney disease; CV, cardiovascular; eGFR, estimated glomerular filtration rate; HF, heart failure; HFp/rEF, HF with preserved/reduced ejection fraction; HHF, hospitalisation for HF; LVEF, left ventricular ejection fraction; T1/2D, type 1/2 diabetes; UACR, urinary albumin–creatinine ratio. Source for study completion dates, prespecified endpoints, enrolment numbers and inclusion criteria: clinicaltrials.gov. *SOLOIST-WHF was terminated early

Recently completed and ongoing dedicated HF and renal studies (Fig. 5A) will provide more evidence on each agent to inform clinical decisions where reducing CV, HF or renal risk is a consideration. Similarly, by including both patients with and without T2D (Fig. 5B, C), these studies suggest that patients without T2D can benefit from certain GLDs where they have a history of HF or CKD [13, 36, 69, 125, 126]—however, evidence from these studies will remain relevant to patients with T2D and their treating physicians, due to the prevalence of comorbid HF and CKD and the CV–renal–metabolic axis [104].

Among ongoing and recently completed HF outcomes trials, studies on patients with HFpEF (Figs. 2A, B and 5C) such as EMPEROR-Preserved phase 3 trial [127], are of particular interest, as no agent of any class has previously shown a clear and unambiguous benefit for this indication [128]. Notably, EMPEROR-Preserved recently met its primary endpoint—empagliflozin significantly reduced the risk of the composite of CV death and HHF in adults with HFpEF > 40%, with or without diabetes [85, 86]. Reductions in the risk of various HF events were observed for inpatients and outpatients [129]. Although empagliflozin appeared to have less of a reno-protective effect in patients with HFpEF than with HFrEF, further analyses of EMPEROR-Preserved indicate that this may be related to the endpoint definition used (which excluded renal death and included ≥ 40% decrease in eGFR), with positive findings when using the renal endpoint from the DAPA-HF trial (which included renal death and ≥ 50% decrease in eGFR) [130, 131]. In an editorial, the author noted that findings for dapagliflozin in the DELIVER trial, in patients with HFpEF > 40%, are also keenly awaited [87].

### Exploring the full potential of SGLT2 inhibitors and GLP-1 RAs: differentiating between clinical trials and the need for real-world evidence

As evidence from renal and HF outcomes studies emerges to add to the wealth of data from CVOTs, the challenge will be to integrate the learnings from an ever-increasing number of studies, and from disparate populations, into clinical practice. Clinicians are faced with untangling many differences in trial design and patient characteristics, and an absence of any direct head-to-head insights. When making evidence-based therapy decisions, it is important to consider trials with relevant study populations, and in particular to bear in mind patients’ diabetes status, as well as CV, HF and renal risk (i.e. factors that should be reflected by licensing approvals for individual medications and up-to-date treatment guidelines). For example, many patients in dedicated HF studies do not have diabetes and, depending on the study, have either HFrEF or HFpEF (Fig. 5C), while patients in the dedicated renal outcomes studies have markedly different renal impairment, albuminuria and diabetes selection criteria between studies (Fig. 5B). This may explain differences seen in outcomes for CV death between some diabetes CVOTs and renal and HF studies. By contrast, HHF outcomes have consistently pointed to a benefit with SGLT2 inhibitors, regardless of the population characteristics. Continuing guideline updates can help clinicians to navigate the commonalities and distinguishing features among the complexity of evidence, such as the current recommendations to distinguish between ASCVD, CKD and HF settings when making treatment decisions in T2D.

To capture cardiorenal outcomes in the full breadth of patients encountered in clinical practice, we may need to look beyond clinical trials to real-world evidence studies, in order to confirm that CVOT findings are consistent in more diverse populations reflective of patients in the clinic [73]. These studies can also help to establish health care resource utilisation benefits, and provide cost implications for the use of SGLT2 inhibitor and GLP-1 RA therapies in everyday practice [132]. Early real-world evidence studies have already begun to confirm a consistent reduction of HHF with SGLT2 inhibitors, and ongoing studies are set to provide more comprehensive insights [73].

The paradigm shift that began with EMPA-REG OUTCOME and LEADER has led to SGLT2 inhibitors and GLP-1 RA being recognised not only in international diabetes guidelines, but also as an important consideration for patients with T2D in CVD prevention [28–30, 76, 77], HF [133–135] and CKD [41, 102] guidelines, where it has been suggested that these agents should be considered early in the course of diabetes management. These developments highlight the shift in treatment goals for T2D, from primarily focusing on the management of hyperglycaemia to a greater appreciation of the importance of managing cardiorenal risk, to reduce the high rates of CV deaths and cardiorenal hospitalisations in patients with T2D. However, SGLT2 inhibitors are not currently approved for primary prevention of cardiorenal comorbidities in T2D; additional evidence on outcomes in this setting may help us to explore the full potential of these agents.

## Conclusions: saving lives with CVOTs

**Fig. 6:**
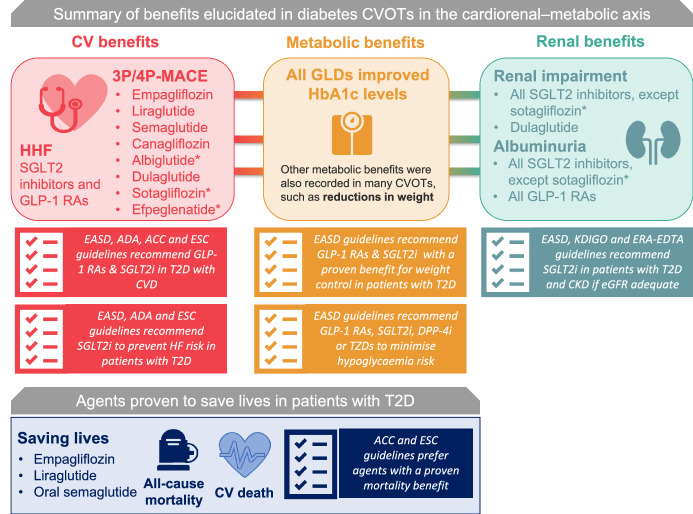
Summary of benefits elucidated in diabetes CVOTs and evolution of international guidelines in light of emerging results. Diabetes CVOTs have enabled professional societies to identify agents that may provide benefits across the cardiorenal–metabolic axis of diabetes; as such, international guidelines have now been updated to reflect the new evidence base represented by these studies. Note that some outcomes suggested a benefit but were not statistically significant due to the ranking of the statistical hierarchy. Not all outcomes have been reported for all agents. 3P-MACE, 3-point major adverse CV event; ACC, American College of Cardiologists; ADA, American Diabetes Association; CKD, chronic kidney disease; CV, cardiovascular; CVD, CV disease; CVOT, CV outcomes trial; EASD, European Association for the Study of Diabetes; ERA-EDTA, European Renal Association-Dialysis and Transplant Association; ESC, European Society of Cardiology; GLP-1, glucagon like peptide-1; HHF, hospitalisation for heart failure; SGLT2i, sodium–glucose transporter 2 inhibitor; T2D, type 2 diabetes. *Albiglutide, sotagliflozin and efpeglenatide are not approved for T2D

CVOTs designed to evaluate the CV safety of GLDs have highlighted clinical findings far greater than might have been originally expected. Providing a plethora of information on potentially unexpected outcomes, they have led to a paradigm shift that began with EMPA-REG OUTCOME and LEADER, and continued with subsequent CVOTs and now HF and renal outcomes studies [13, 60]. Despite the underlying mechanisms of such findings remaining a matter of theoretical postulation [60, 110–112, 124], the contribution of CVOTs as new evidence to the diabetes treatment armamentarium highlights a new era of standard treatment practices; endorsed by international guidelines such as ADA and EASD, to highlight the potential of SGLT2 inhibitors and GLP-1 RAs to improve cardiorenal outcomes for patients with T2D [38, 40]. In the post-CVOT era, people living with T2D are now able to benefit from treatments that can provide a therapeutic effect across the cardio-renal metabolic axis of T2D, while their physicians have options to achieve clinically meaningful reductions in CV, HF and renal outcomes, and even to reduce mortality (Fig. 6).

## Supplementary Information

**Additional file 1: Table S1.** Expanded details of CVOTs, renal and HF studies in patients with T2D. Overview of diabetes CVOTs, renal outcomes studies and HF studies with glucose-lowering drugs in patients with T2D, including key outcomes and baseline characteristics [15, 16, 18, 20–22, 26–37, 49–55, 57–59, 62, 63, 68–72, 92, 99, 125].

**Additional file 2: Figure S1.** Continuum of CV risk in T2D. T2D is a risk factor for CVD, and several other risk factors are also often present in patients with T2D, as recognised by guidelines such as those of the ESC [38]. Glucose levels alone can be independently linked to progression of CAD [38]. While progression of cardiac disease is thus a feature of T2D, it may in some cases go undetected due to atypical symptom presentation or so-called ‘silent’ manifestations [152, 153], in the proposed ‘unrecognised diabetic cardiac impairment’ phenomenon [48]. Ultimately, overt CVD or heart failure may develop, both of which are prevalent among people living with T2D [4, 48, 152]. CAD, coronary artery disease; CVD, cardiovascular disease; ESC, European Society of Cardiology; MACE, major adverse cardiovascular events; T2D, type 2 diabetes. **Figure S2.** What to expect next from CVOT-related research. The results of CVOTs have raised several questions that are now being addressed in clinical and scientific studies, chief among which is: how do glucose-lowering drugs produce glucose-independent beneficial effects on cardiorenal outcomes? CVOT, cardiovascular outcomes trial; GLP-1, glucagon like peptide-1; GLP-1 RA, GLP-1 receptor agonist; SGLT2, sodium–glucose transporter 2.

## Data Availability

Not applicable—new data or materials were used for this manuscript.

## Abbreviations

ACC: American College of Cardiology
ADA: American Diabetes Association
ASCVD: Atherosclerotic cardiovascular disease
BMI: Body mass index
CI: Confidence interval
CV: Cardiovascular
CVD: Cardiovascular disease
CVOT: Cardiovascular outcomes trial
DPP-4: Dipeptidyl peptidase-4
EASD: European Association for the Study of Diabetes
EDTA: European Dialysis and Transplant Association
eGFR: Estimated glomerular filtration rate
ERA: European Renal Association
ESC: Europe Society of Cardiology
ESRD: End-stage renal disease
EURECA-m: European Renal and Cardiovascular Medicine
FDA: U.S. Food and Drug Administration
GLD: Glucose-lowering drug
GLP-1 RA: Glucagon-like peptide-1 receptor agonist
Hb1Ac: Haemoglobin A1c
HF: Heart failure
HFpEF: Heart failure with preserved ejection fraction
HFrEF: Heart failure with reduced ejection fraction
HHF: Hospitalisation for heart failure
HR: Hazard ratio
KDIGO: Kidney Disease Improving Global Outcomes
MACE: Major adverse cardiovascular events
MI: Myocardial infarction
NR: Not reported
RRR: Relative risk reduction
SCr: Serum creatinine
SGLT2: Sodium–glucose transporter 2
T2D: Type 2 diabetes
TIMI: Thrombolysis In Myocardial Infarction
3P-MACE: 3-Point major adverse cardiovascular events
4P-MACE: 4-Point major adverse cardiovascular events
