# Supplementary material for: Cardiovascular outcomes trials: a paradigm shift in the current management of type 2 diabetes
Source: Cardiovasc Diabetol. 2022 Aug 4;21:144. doi: 10.1186/s12933-022-01575-9 (PMC9351217; doi:10.1186/s12933-022-01575-9)
Supplement: Supplementary file 2 — Additional file 2: Figure S1. Continuum of CV risk in T2D. T2D is a risk factor for CVD, and several other risk factors are also often present in patients with T2D, as recognised by guidelines such as those of the ESC [38]. Glucose levels alone can be independently linked to progression of CAD [38]. While progression of cardiac disease is thus a feature of T2D, it may in some cases go undetected due to atypical symptom presentation or so-called ‘silent’ manifestations [152, 153], in the proposed ‘unrecognised diabetic cardiac impairment’ phenomenon [48]. Ultimately, overt CVD or heart failure may develop, both of which are prevalent among people living with T2D [4, 48, 152]. CAD, coronary artery disease; CVD, cardiovascular disease; ESC, European Society of Cardiology; MACE, major adverse cardiovascular events; T2D, type 2 diabetes. Figure S2. What to expect next from CVOT-related research. The results of CVOTs have raised several questions that are now being addressed in clinical and scientific studies, chief among which is: how do glucose-lowering drugs produce glucose-independent beneficial effects on cardiorenal outcomes? CVOT, cardiovascular outcomes trial; GLP-1, glucagon like peptide-1; GLP-1 RA, GLP-1 receptor agonist; SGLT2, sodium–glucose transporter 2. [file 12933_2022_1575_MOESM2_ESM.pptx]

## Slide 1
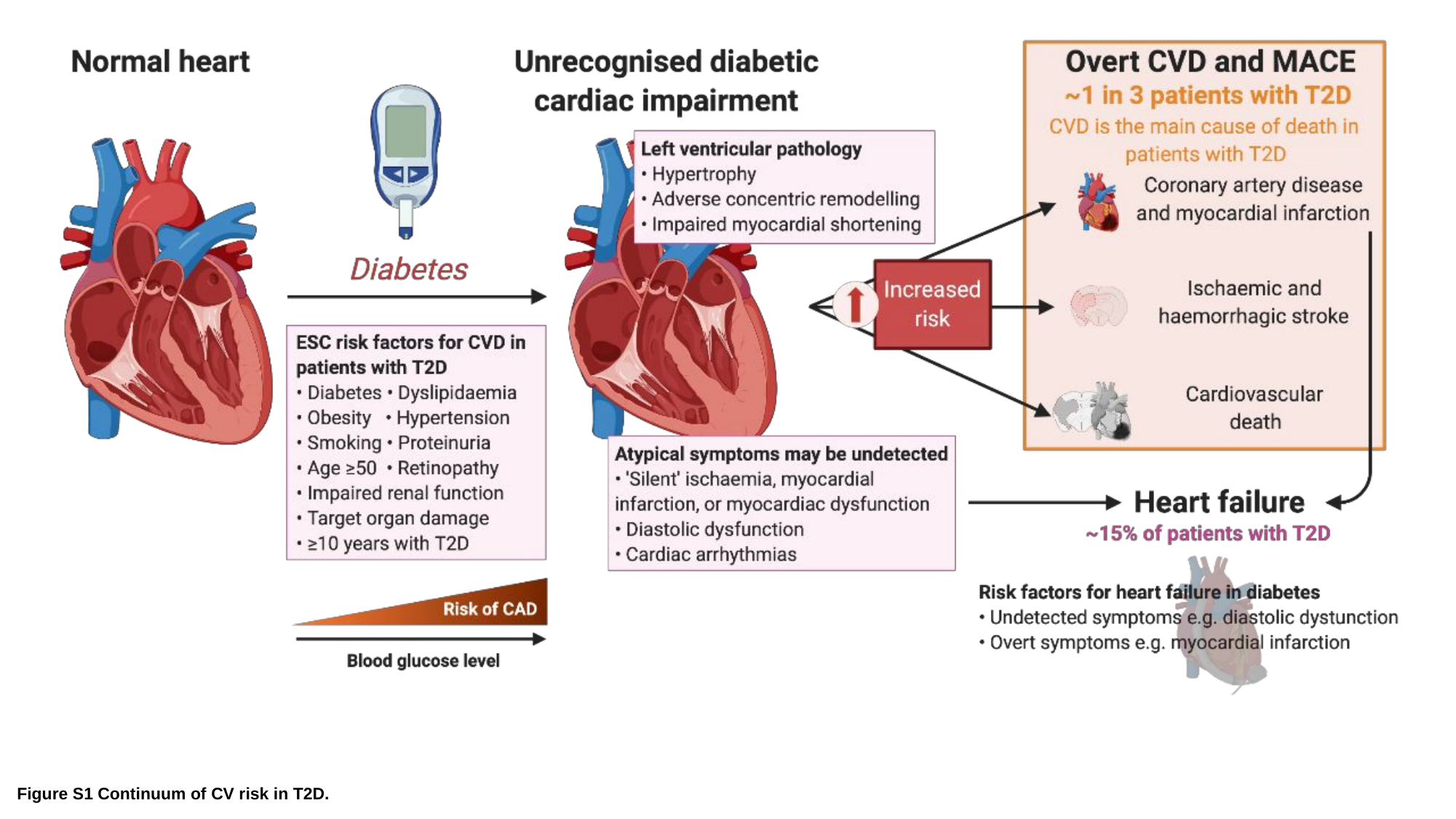

Figure S1 Continuum of CV risk in T2D.

## Slide 2
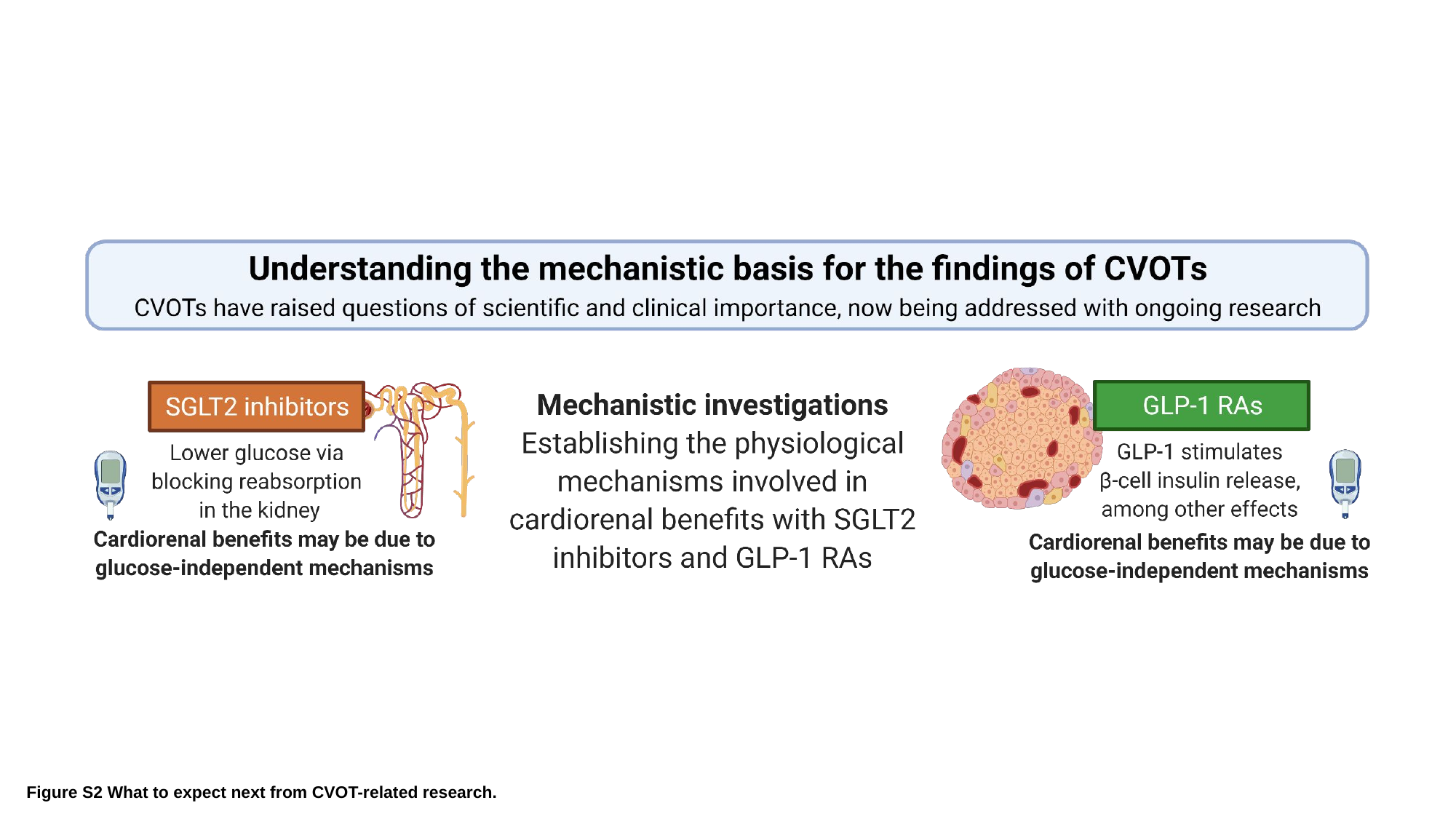

Figure S2 What to expect next from CVOT-related research.
